# When Attention Fails Memory: Voluntary Orienting Deficits and Visual Short-Term Memory in Intellectual Disability

**DOI:** 10.5334/joc.498

**Published:** 2026-04-20

**Authors:** Andria Shimi, Christina Charalambidou

**Affiliations:** 1 Department of Psychology, University of Cyprus, Cyprus

**Keywords:** intellectual disability, visuospatial selective attention, attentional orienting, visuospatial short-term memory, fluid intelligence, non-verbal reasoning

## Abstract

Individuals with intellectual disability (ID) exhibit deficits in both selective attention and visual short-term memory (VSTM). However, whether attentional deficits directly constrain VSTM performance in ID remains unknown. Here, we examined the interplay between these two processes in ID and their correlates with fluid intelligence across individuals with varying levels of intellectual functioning. Nineteen adults with ID and twenty-two chronologically matched neurotypical adults (TDA) carried out the Attentional Orienting Task (AOT), in which they briefly viewed a memory array, followed by a probe and indicated whether the probe was previously presented in the memory array. Visuospatial attention cues were shown before (pre-cues) or after (retro-cues) the memory array to assess attentional orienting in service of VSTM encoding and maintenance, respectively, compared to neutral, baseline attention cues. Additionally, participants completed Raven’s 2, a non-verbal reasoning test. TDA adults outperformed individuals with ID across all conditions. Importantly, while TDA demonstrated clear attentional orienting benefits in service of VSTM, individuals with ID showed no cueing benefits either before encoding into VSTM or during VSTM maintenance. Correlation and regression analyses showed that attentional orienting abilities predicted fluid intelligence beyond baseline VSTM performance. Current results show for the first time that attentional deficits constrain VSTM performance in ID, whereas individual differences in attentional orienting in service of VSTM predict non-verbal reasoning abilities. These findings speak to the overall functioning difficulties individuals with ID face and highlight the need to consider the dynamic relation between these cognitive processes when designing assessments and interventions for intellectual disabilities.

## Introduction

Visual short-term memory (VSTM) is a limited-capacity (Cowan, 2001) foundational cognitive system that allows individuals to retain visual information briefly. It is essential for various everyday cognitive operations, from simple perceptual judgments to advanced reasoning and goal-directed actions (Schurgin, 2018). Although the term VSTM is often used interchangeably with visual working memory [VWM; (Oberauer et al., 2018)], we use VSTM here to emphasise short-term storage in the absence of dual-task processing requirements (Logie, 2011). Among the core processes supporting VSTM are attentional mechanisms that enable the selection, encoding, and active maintenance of task-relevant information (Chun & Johnson, 2011; Gazzaley & Nobre, 2012; Oberauer, 2019). Yet, despite this growing understanding in neurotypical populations, less is known about how attentional mechanisms constrain VSTM in individuals with intellectual disability (ID), a population characterised by persistent cognitive challenges across multiple domains. The current study addresses this gap by examining whether adults with ID orient attention to support encoding and maintenance in VSTM. Understanding whether VSTM impairments in ID arise from capacity constraints, inefficient encoding or maintenance, remains an open and important question, one with implications for both theory and intervention.

Intellectual Disability (ID) is a neurodevelopmental condition characterised by significant limitations in intellectual functioning and adaptive behaviour (American Psychiatric Association, 2022), which interfere with daily functioning and learning (Schalock et al., 2021). Severity levels of ID are specified as mild, moderate, severe, and profound. Although these levels rely primarily on adaptive functioning, typical descriptive IQ scores range between 50–70 for mild ID, 35–50 for moderate ID, 20–35 for severe ID, and below 20 for profound ID. Individuals with mild ID are often relatively independent in daily living skills, but experience marked abstract reasoning and academic difficulties, whereas those with moderate ID require more structured support, show more pronounced limitations in conceptual functioning, with academic skills typically remaining at an elementary level. Severe and profound ID are characterised by greater impairments in conceptual, social, and practical domains and typically require pervasive support across daily activities. Individuals with ID represent a heterogeneous group, with cognitive profiles differing widely depending on severity and aetiology. Syndromic ID is often associated with identifiable chromosomal, metabolic, or environmental causes (Leonard & Wen, 2002; Vissers et al., 2016), whereas non-syndromic ID lacks a known underlying cause and presents primarily with global cognitive deficits (Ropers & Hamel, 2005). Although aetiologies vary, many forms of ID share impairments in core cognitive systems.

Specifically, individuals with ID experience pronounced and pervasive impairments in selective attention, the ability to prioritise relevant over irrelevant information, and this limitation has long been posited as a core source of broader cognitive delays (Crosby & Blatt, 1968; Cruse, 1961; Zeaman & House, 1963). Compared with typically-developing peers, individuals with ID are markedly more susceptible to distraction (Brown, 1966; Burack, 1994), show reduced efficiency in filtering irrelevant information (Hagen & Huntsman, 1971; Merrill, 2006; Merrill et al., 1994) and allocate processing resources less effectively under increasing cognitive load (Ellis & Dulaney, 1991; Merrill & Peacock, 1994; Nugent & Mosley, 1987; Oka & Miura, 2008). Such attentional impairments have been linked to challenges in everyday functioning and reduced independence (García-Pintor et al., 2024), highlighting their cascading effects in other domains. While such findings were once attributed to a global reduction in attentional capacity, converging evidence now indicates a more specific weakness in the voluntary (endogenous) orienting of attention. Indeed, recent reviews have challenged the notion of a global attentional deficit in ID, instead highlighting the need for developmentally informed, task-specific analyses (Burack et al., 2023). In spatial cueing and other attentional control paradigms, individuals with ID derive smaller benefits from symbolic cues, shift attention more slowly to cued locations, and struggle to disengage from irrelevant stimuli, suggesting a deficit in the mechanisms that guide the intentional allocation of spatial attention (Boot et al., 2013; Kim et al., 2021; Merrill & O’Dekirk, 1994).

VSTM is another domain that is often impaired in ID. Individuals with ID show markedly reduced VSTM capacity in simple span tasks across aetiologies (Henry & MacLean, 2002; Vicari et al., 1995). Their poor performance in VSTM tasks is not only due to reduced capacity but also due to inefficient encoding strategies and heightened vulnerability to interference during visual memory tasks (Henry & MacLean, 2002). Moreover, difficulties in maintaining bound representations, such as the conjunction of item identity and location, have been reported in ID-associated syndromes (Jarrold & Brock, 2012), pointing to deficits beyond storage limitations alone. In short, while certain syndromes show relative strengths or weaknesses in specific modalities (Lanfranchi et al., 2004; Vicari & Carlesimo, 2006), overall performance in VSTM tasks remains markedly lower than in typically developing individuals, even after matching for mental age (Henry & MacLean, 2002; Lanfranchi et al., 2009; Vicari & Carlesimo, 2006).

Taken together, findings from these two cognitive domains support the view that both selective attention and VSTM are compromised in ID. Notably, memory impairments in ID may stem in part from an inability to recruit attentional orienting efficiently during encoding or maintenance, rather than from reduced capacity alone. This is consistent with broader evidence that individual differences in VSTM capacity reflect differences in attentional orienting, rather than storage per se, both in neurotypical adults (Fukuda & Vogel, 2009; Murray et al., 2011; Vogel et al., 2005) and children (Shimi et al., 2015; Shimi, Kuo, et al., 2014; Shimi, Nobre, et al., 2014; Shimi & Scerif, 2015) and suggests that attentional dysfunction may play a causal role in the memory difficulties observed in ID.

In neurotypical adults, visuospatial attentional orienting and VSTM are functionally interdependent; that is, attentional orienting modulates the encoding and maintenance of relevant items into VSTM through top-down mechanisms (Van Ede & Nobre, 2023). Spatial cueing paradigms have been pivotal in demonstrating this relation. Pre-cues, presented before stimulus onset, allocate attention prospectively to the relevant perceptual input, whereas retro-cues, presented during maintenance, direct attention internally toward specific items already stored in VSTM. Both cue types yield robust behavioural benefits, improving adults’ performance (Griffin & Nobre, 2003; Lepsien et al., 2011; Matsukura et al., 2007), reflecting the flexible and goal-directed nature of attention in service of VSTM. Computational modelling with adults (Hollingworth & Hwang, 2013; Makovski & Pertzov, 2015; Souza et al., 2016), and developmentally (Shimi & Scerif, 2022) has shown that attentional orienting in service of VSTM can increase the likelihood of correct item selection, reduce misbinding errors, and lower the probability of random guessing, as well as protect from interference during maintenance (Shimi, Nobre, et al., 2014), processes already documented as impaired in ID.

Developmental work has further revealed important age-related changes in the interplay between selective attention and VSTM. While even children as young as 6 years old can benefit from pre-cues, indicating an early-emerging ability to focus attention prospectively, their capacity to deploy attention retrospectively develops more slowly, with reliable benefits typically emerging later in childhood (around 10–12 years), and remains highly variable throughout middle childhood (Shimi, Nobre, et al., 2014; Shimi & Scerif, 2015, 2017).

Importantly, attentional control has also been linked to individual differences in fluid reasoning. In neurotypical populations, measures of attentional control and VSTM/VWM capacity reliably predict adult (Engle et al., 1999; Heitz et al., 2005) and child (Cowan et al., 2006) performance on non-verbal intelligence tasks, pointing to a close relation between attentional control, memory processes, and fluid reasoning. Thus, examining whether attentional orienting efficiency explains unique variance in non-verbal reasoning across individuals with varying levels of intellectual functioning provides a dimensional evaluation of how domain-general attentional orienting mechanisms relate to broader cognitive ability.

Building on the developmental evidence that improvements in VSTM rely heavily on maturing attentional control, the present study extends this framework to ID. If attentional control mechanisms are compromised, as documented in individuals with ID, we would expect reduced benefits from attentional cues that require intentional prioritisation of perceptual input and/or voluntary attentional allocation to internal representations. By manipulating attentional orienting at encoding (pre-cues) and during maintenance (retro-cues), we directly tested whether VSTM impairments in ID reflect reduced storage capacity per se and/or reduced efficiency in deploying voluntary attention at distinct processing stages. We hypothesised that the VSTM difficulties observed in ID arise primarily from limitations in voluntary attentional orienting, especially during the maintenance phase, rather than from storage deficits alone. We predicted three outcomes: (1) overall cueing benefits would be smaller in the ID group, consistent with a broad impairment in voluntary attention; (2) the reduction in cueing benefits would be strongest for retro-cues, compared to pre-cues, consistent with developmental evidence that retrospective attentional orienting emerges later and remains more variable, suggesting particular difficulty allocating attention to internal representations that place greater demands on voluntary control mechanisms; and (3) the efficiency of attentional orienting would account for unique variance in non-verbal reasoning, over and above basic VSTM capacity. To the best of our knowledge, this is the first study to directly evaluate whether deficits in external and internal attention, rather than limited storage, drive VSTM impairments in ID.

In the present study, we focused on individuals with ID who varied in aetiology but shared a diagnosis of mild to moderate ID. Although the sample was clinically heterogeneous, prior literature has consistently documented that individuals with ID are at increased risk for deficits in attentional control and VSTM, as already stated. Our sampling strategy reflects a transdiagnostic, dimensional approach in neurodevelopmental research, which emphasises the study of core cognitive processes, such as selective attention and VSTM, which are implicated across different aetiological subgroups (Astle & Fletcher-Watson, 2020; Burack et al., 2021). By adopting this mechanism-oriented perspective, the current study examined attentional orienting as a potential source of VSTM impairments in ID, irrespective of aetiological origin. This approach allows for a more precise characterisation of cognitive functioning in ID and supports the development of interventions targeting shared neurocognitive vulnerabilities.

## Methods

### Participants

Twenty-five adults with ID (ID group henceforth; aged 19–63 years) and twenty-two chronologically matched neurotypical adults (TDA group henceforth; aged 19–62 years) voluntarily participated in the study. Participants with ID were recruited through a national foundation that provides care, employment, and therapeutic services to individuals with mild to moderate ID. Inclusion criteria for the ID group were: (1) age 18 years or older, (2) clinical diagnosis of mild to moderate ID, (3) ability to communicate verbally, and (4) absence of severe motor or visual-auditory impairments. Inclusion criteria for the TDA group were: (1) chronological age within the ID age range, (2) no history of neurological conditions, and (3) no severe motor or visual-auditory impairments. All participants had normal or corrected-to-normal vision.

Of the 25 participants initially recruited to the ID group, six (2 males, 4 females) did not complete both cognitive tasks and were excluded from further analyses. The final ID sample consisted of 19 participants (8 males, 11 females; Mage = 39.47, SD = 13.52), including five individuals aged 19–29, five aged 30–40, five aged 45–48, and four aged 54–63. The TDA group included 22 participants (11 males, 11 females; Mage = 39.18, SD = 13.76), with nine aged 19–29, one aged 30, seven aged 42–49, and five aged 52–62. Based on the provided consent, diagnostic information was available for 15 out of the 19 participants. Of these, two had syndromic ID, while the remaining had non-syndromic ID, with or without co-occurring conditions. Excluding participants with syndromic ID did not alter the results.

The sample size was constrained by the available numbers at the National Foundation, the number of returned consent forms, and the feasibility of recruiting and testing adults with ID in an experimental paradigm requiring sustained task engagement and conceptual understanding. We also carried out power analyses to ensure sufficient statistical power (see Statistical Design).

The study received ethical approval from the Cyprus National Bioethics Committee and adhered to the principles of the Declaration of Helsinki. All TDA participants and the parents or legal guardians of individuals with ID provided written informed consent. Additionally, all participants with ID provided verbal assent before participation.

### Apparatus

#### Attentional Orienting Task (AOT)

**Figure 1:**
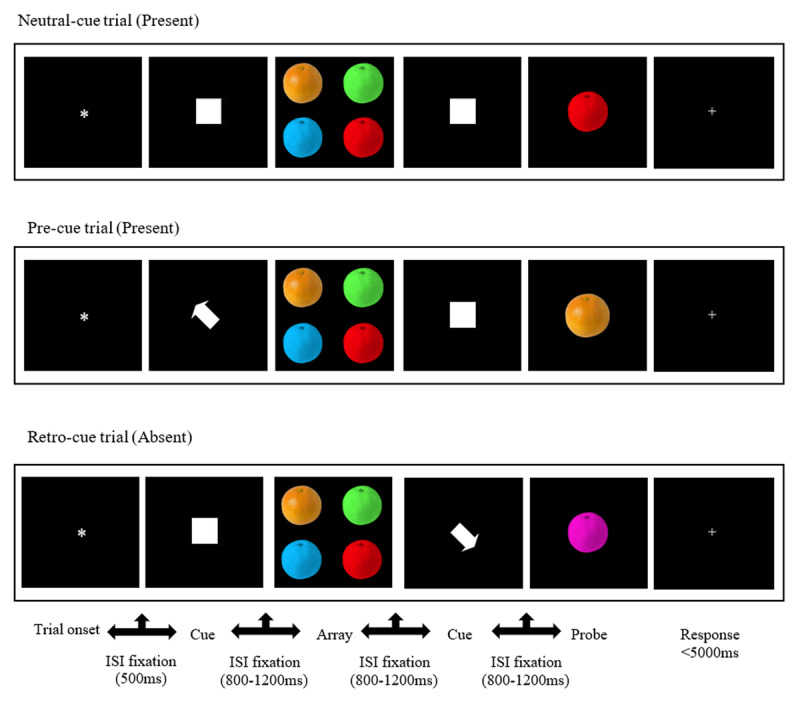
Schematic illustration of the task and the three different types of trials.

The task was identical to that used in developmental research (Shimi, Kuo, et al., 2014; Shimi, Nobre, et al., 2014; Shimi & Scerif, 2017). In each trial, participants viewed briefly (350 ms) a memory array consisting of four coloured items, presented on a black background. Items were symmetrically arranged around a central fixation point, with one item centred in each quadrant (eccentricity: 2.87° visual angle horizontally and vertically). Each item subtended approximately 1.64° × 2.05° of visual angle and was coloured in one of seven hues (blue, green, yellow, orange, magenta, red, or white). Following a variable delay (all inter-stimulus intervals: 800–1200 ms), a single coloured probe item appeared centrally (350 ms), and participants responded via button press to indicate whether the probe had been part of the preceding memory array (left button for “present,” right for “absent”). The task was presented with E-Prime 2.0 (Psychological Software Tools, Inc., Pittsburgh, PA) on a laptop computer. A schematic illustration of the task is presented in Figure 1.

In half of the trials, attentional orienting was manipulated using visuospatial cues (presented for 300 ms each). In pre-cue trials, a centrally presented white arrow (0.82° × 0.82°) appeared before the memory array and indicated which of the upcoming items should be prioritised during encoding. In retro-cue trials, a white arrow appeared after the memory array and directed attention internally to one of the items that had already been encoded in VSTM (i.e., during maintenance). All spatial cues were 100% valid in probe-present trials, always indicating the location of the item that would later be tested, if it had appeared in the memory array. Neutral trials included centrally presented white-filled squares (uninformative cues) shown before and after the array to control for non-spatial alerting effects. To further equate alerting across conditions, each pre-cue trial was followed by a neutral square at the retro-cue time point, and each retro-cue trial was preceded by a neutral square at the pre-cue time point.

The task began with two practice blocks of six trials each. The first practice block used slower timings to facilitate understanding, especially for participants with ID, in line with procedures used in young child testing (Shimi, Nobre, et al., 2014; Shimi & Scerif, 2015, 2017, 2022), while the second matched the timing of the experimental trials. This two-stage procedure ensured that participants in the ID group comprehended the task and transitioned smoothly from the slower to the faster timing trials. Participants then completed four test blocks of 48 trials each, totalling 192 experimental trials: 128 probe-present (67%) and 64 (33%) probe-absent trials. Half of all trials were spatially cued (with equal probability to cue one of the four possible locations) and half were neutral. Of the probe-present trials, 32 were pre-cue trials, 32 were retro-cue trials, and 64 were neutral trials. Of the probe-absent trials, 16 were pre-cue trials, 16 were retro-cue trials, and 32 were neutral trials. Spatial cues appeared in both probe-present and probe-absent trials to prevent the cue from becoming predictive of a “present” response. In probe-absent trials, the spatial cue indicated the location of an item that had been present in the array, but the probe did not match any array item. The task was divided into two test blocks including pre-cue and neutral trials, and two test blocks including retro-cue and neutral trials to minimise potential confusion for participants with ID. Block order alternated and was counterbalanced across participants. Cued and neutral trials were randomly intermixed within each block.

Participants received visual and oral feedback on each practice trial (i.e., “correct”, “incorrect”, “no response”), whereas, in the experimental phase, cumulative performance feedback was provided after every 16 trials and again at the end of each block. Self-paced breaks were included every 16 trials within each block and between test blocks.

#### Raven’s 2

Non-verbal reasoning ability was assessed using the Raven’s 2 European Standardisation (Raven, 2019), a widely used measure of general cognitive functioning suitable for individuals aged 4 to 69 years. Its non-verbal format makes it particularly appropriate for individuals with limited verbal communication or from special populations, including those with ID. The test primarily measures fluid reasoning, inductive thinking, and the ability to identify abstract patterns and relationships, while also drawing on related processes such as visual attention, spatial perception, working memory, and classification (McLeod & McCrimmon, 2021).

Raven’s 2 consists of 60 matrix-based items grouped into five sets (A–E), each comprising 12 colourful geometric matrices arranged in varying layouts (e.g., 2 × 2, 3 × 3, 1 × 6). Each matrix is missing one element that participants are asked to complete by selecting one item from six alternatives. In line with standardised guidelines, participants with ID completed the short version (Sets A-C) with a 30-minute time limit, while TDA participants completed the full version (Sets B-E) with a 45-minute limit. Test administration was considered valid if the participant completed at least 16 matrices, discontinued based on standard stopping rules, or reached the allotted time. All included participants met these criteria and had a valid test administration.

### Procedure

Participants with ID were tested individually in a quiet room within the foundation, whereas TDA participants were tested in the lab at the university. All participants sat at a comfortable viewing distance from the screen. The experimenter introduced the AOT using example trials presented on cards and explained the different trial types. Participants were instructed to attend to the spatial cues, when present, as these would help them determine whether the probe had appeared in the preceding memory array. To ensure that participants with ID understood the function of spatial cues, they were also given paper-based examples and asked to explain how they would respond to pre-cues and retro-cues. Only those who demonstrated a clear understanding of the purpose of the spatial cues, and, by extension, the AOT, were included in the final sample. Participants with ID placed the index finger of each hand on each mouse button [consistent with procedures used in AOT child testing; (cf. Shimi, Nobre, et al., 2014; Shimi & Scerif, 2015, 2017), whereas TDA participants held the mouse with one hand. Finally, during the practice phase, the experimenter provided verbal feedback to ensure that participants understood the task requirements.

Following completion of the AOT, TDA participants took a five-minute break before completing Raven’s 2 in the same session. To minimise fatigue, participants with ID completed Raven’s 2 on a separate day at the foundation. Before test administration, all participants completed a sample matrix item with experimenter guidance, as per standardised procedures, and received corrective feedback to confirm task understanding. During the test, participants with ID indicated their responses by pointing to their chosen option, and the experimenter recorded the answers on the response sheet. TDA participants completed the response sheet independently.

### Statistical Design

A mixed-design Analysis of Variance (ANOVA) was conducted on mean d-prime (d′), a sensitivity-based measure of response accuracy. The within-subject variables were cue-condition (pre-cue, retro-cue) and trial-type (cued, neutral), and the between-subjects variable was group (ID, TDA). Cue-condition (pre-cue, retro-cue) signified the stage at which attentional orienting was manipulated (encoding vs maintenance), whereas trial-type (cued, neutral) indicated whether informative attentional cues were available relative to the stage-matched neutral baseline. Neutral trials were analysed separately within pre-cue and retro-cue blocks to provide baseline comparisons for each orienting stage. Bonferroni corrections were employed when necessary. d′ is a sensitivity-based measure of response accuracy, reflecting the participant’s ability to discriminate whether the probe had appeared in the preceding array. It was calculated using the formula: d′ = z (hit rate) – z (false alarm rate). Extreme scores for hit and false alarm rates were corrected using the formulas 1/(2 N) and 1 – (1/2 N), as recommended by Macmillan and Creelman (2005), where N refers to the number of total trials in a given condition.

To complement the frequentist analyses and to quantify the strength of evidence for the presence or absence of effects, Bayesian analyses were conducted in JASP (version 0.19.3) using default priors. Specifically, a Bayesian mixed-design ANOVA corresponding to the frequentist model was performed. Evidence for each effect was evaluated using inclusion Bayes factors, which quantify the relative likelihood of models including versus excluding a given effect. Bayes factors greater than 30 are interpreted as very strong evidence. Follow-up Bayesian paired-samples t-tests were conducted within the ID group to quantify the strength of evidence for the absence of cueing benefits. Bayes factors favouring the null hypothesis (BF_01_) were calculated, allowing us to distinguish between a lack of evidence and evidence supporting the null hypothesis, with BF_01_ values above 3 interpreted as moderate evidence for the null.

Reaction time (RT) analyses were not conducted, as a subset of participants in the ID group exhibited low accuracy in certain cue-condition × trial-type combinations, precluding reliable RT calculation across all conditions.

To assess whether individual differences in attentional orienting efficiency were associated with overall cognitive ability across the ability spectrum, Pearson’s r correlations were conducted across the full sample (ID and TDA combined) between raw Raven’s 2 scores and d′, followed by hierarchical linear regressions to determine whether performance in the cued conditions explained unique variance in non-verbal reasoning beyond baseline VSTM performance.

To ensure that our sample sizes for individuals with ID (N = 19) and TDA (N = 22) afforded sufficient statistical power, we carried out a power analysis using G*Power 3.1 (Faul et al., 2007). Based on published developmental studies employing the same attentional orienting paradigm (Shimi, Nobre, et al., 2014; Shimi & Scerif, 2017), the lowest reported partial eta squared value for the critical three-way interaction (Group × Cue-condition × Trial-type) was η_p_^2^ = .17 (corresponding to f = 0.45). The analysis indicated that the current sample (N = 41) provided very high power to detect effects of this magnitude (power > .99, α = .05).

## Results

### D-prime

Analyses revealed statistically significant main effects of cue-condition [F(1, 39) = 7.33, p = .01, η_p_^2^ = .16)], trial-type [F(1, 39) = 99.73, p < .001, η_p_^2^ = .72] and group [F(1, 39) = 172.45, p < .001, η_p_^2^ = .82]. There were, also, statistically significant two-way interactions between cue-condition × group [F(1, 39) = 4.94, p = .03, η_p_^2^ = .11] and trial-type × group [F(1, 39) = 81.28, p < .001, η_p_^2^ = .68]. Importantly, the three-way interaction of cue-condition × trial-type × group was significant [F(1, 39) = 7.89, p = .008, η_p_^2^ = .17].

**Figure 2:**
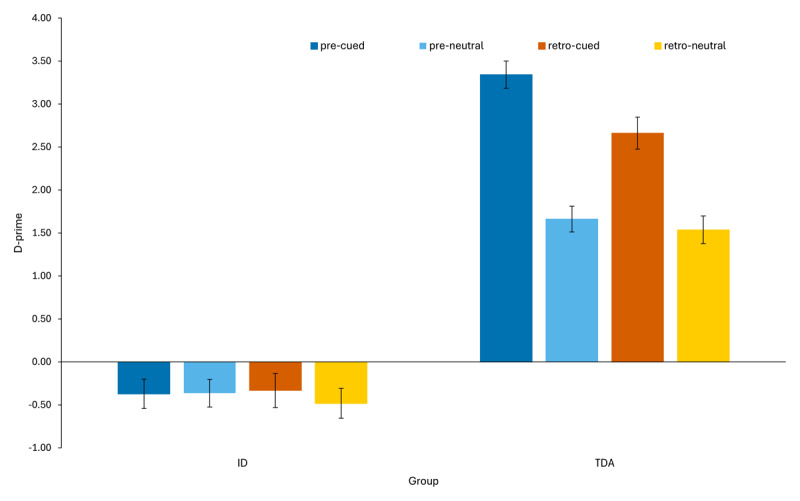
d’ scores for cued and neutral trials, comparing cue conditions, for the ID and TDA groups. Error bars represent standard errors of the mean.

Analyses of simple main effects for the three-way interaction (Figure 2) showed that the TDA group had higher d′ scores than the ID group across both cue conditions, pre-cue and retro-cue, and across trial-types, cued and neutral trials (TDA: M = 3.34 for pre-cue, M = 1.66 for neutral trials in the pre-cue condition, M = 2.66 for retro-cue, M = 1.54 for neutral trials in the retro-cue condition; ID: M = –.37 for pre-cue, M = –.36 for neutral trials in the pre-cue condition, M = –.33 for retro-cue, M = –.48 for neutral trials in the retro-cue condition, all ps < .001). Importantly, the TDA group had higher d′ scores in cued than in neutral trials in both pre-cue (p < .001) and retro-cue conditions (p < .001), indicating clear pre-cue and retro-cue benefits. In contrast, the ID group showed no significant differences between cued and neutral trials in either pre-cue (p = .96) or retro-cue (p = .24) conditions, indicating the absence of cueing benefits. Finally, the TDA group had higher d′ scores in the cued trials of the pre-cue condition than in the cued trials of the retro-cue condition (p < .001), whereas their performance between the neutral trials across the two cue conditions did not differ (p = .26), suggesting larger pre-cue than retro-cue benefits. The ID group showed no significant differences in d′ performance between pre-cue and retro-cue conditions for either cued (p = .83) or neutral trials (p = .32). The pattern of results remained unchanged when age was included as a covariate. Condition-level accuracy rates for the AOT are reported in the Supplementary Material (Table S1).

Subsequent analyses of difference scores (pre-cue – pre-neutral; retro-cue – retro-neutral) revealed that the TDA group had larger cueing benefits than the ID group in both pre-cue and retro-cue conditions, t(39) = –7.83, p < .001 and, t(39) = –5.69, p < .001, respectively, indicating that the group differences reflect differences in attentional orienting rather than overall differences in baseline sensitivity.

The Bayesian ANOVA provided very strong evidence for the critical three-way interaction (BF_inclusion = 53.51), indicating that models including the interaction were over 50 times more likely than models excluding it. Importantly, the model including all main effects and interactions provided the best fit to the data (BF_10_ = 1.57 × 10^27^ relative to the null model). Thus, the Bayesian results converge with the frequentist ANOVA in supporting differential cueing effects across groups and cue conditions.

Follow-up Bayesian paired-samples t-tests within the ID group provided moderate (BF_01_ = 4.20) and anecdotal (BF_01_ = 2.61) evidence in favour of the null hypothesis over the alternative in the pre-cue and retro-cue conditions, respectively, suggesting an absence of d′ cueing benefits and further corroborating our frequentist results.

### Correlations

Pearson’s r correlation analyses were carried out between the participants’ total raw scores on Raven’s 2 and the d’ scores for the different trial types (pre-cued, retro-cued, pre-neutral, and retro-neutral) to examine the overall association between attentional orienting, VSTM baseline performance, and non-verbal ability. It should be noted that the total scores of participants with ID on Raven’s 2 were lower than those of the general population, with their estimated IQ scores falling within the mild to moderate intellectual disability range, consistent with their diagnosed level of impairment. None of the individuals in the TDA group scored in the ID range. The total raw score in Raven’s 2 was used as a continuous variable. Descriptive statistics for Raven’s 2 raw scores are provided in the Supplementary Material (Table S1). The analyses showed very strong positive correlations (Table 1) between the total raw score in Raven’s 2 and pre-cued, retro-cued, pre-neutral, and retro-neutral trials (all ps < .001). Furthermore, partial correlations between Raven’s 2 scores and performance on cued trials (pre-cue and retro-cue conditions), controlling for neutral baseline performance, showed again highly significant positive correlations for both pre-cue, r(38) = .77, p < .001, and retro-cue conditions, r(38) = .50, p < .001. This latter analysis offers a more stringent test of the relationship, as it isolates the contribution of attentional orienting while accounting for general VSTM demands that are also involved in performing Raven’s 2 matrices.

**Table 1:** Relations between attentional orienting measure (d’) and raw scores for non-verbal ability (Raven’s 2).


	d′ PRE-CUED	d′ RETRO- NEUTRAL	d′ RETRO-CUED	RAVEN’S 2

d′ pre-neutral	.88***	.92***	.90***	.85***

d′ pre-cued		.87***	.91***	.94***

d′ retro-neutral			.93***	.82***

d′ retro-cued				.85***


*p < .05, **p < .01, ***p < .001.

It should be noted that age did not correlate with either AOT d′ scores (ps > .20) or Raven’s 2 performance (p = .77). Notably, all results remained unchanged when age was included as a covariate, indicating that age was not a confounding factor in the current analyses.

Finally, we conducted hierarchical linear regressions to assess whether attentional orienting performance in cued trials predicted Raven’s scores after controlling for baseline VSTM performance. Neutral trials were entered in Step 1 to control for general VSTM accuracy and cued trials were entered in Step 2. This approach paralleled the partial correlation analyses and allowed us to evaluate the unique contribution of attentional cueing. Given the theoretical and neurocognitive distinction between cue conditions (Chun et al., 2011; Nobre & Gresch, 2025; Shimi, Kuo, et al., 2014; Shimi, Nobre, et al., 2014; Shimi & Scerif, 2017; Van Ede & Nobre, 2023), separate regression models were conducted for pre-cue and retro-cue conditions to examine whether encoding-related (pre-cue) versus maintenance-related (retro-cue) attention predicted fluid intelligence differently. For the pre-cue condition, neutral d′ significantly predicted Raven’s scores in Step 1, *β* = .85, p < .001, accounting for 71.6% of the variance (*R*^2^ = .72). In Step 2, adding pre-cued d′ significantly improved the model, Δ*R*^2^ = .17, F(1, 38) = 56.84, p < .001, with pre-cued d′ emerging as a strong independent predictor, *β* = .88, p < .001. Collinearity diagnostics indicated no concerns (VIF = 4.53; Tolerance = .22). In contrast, neutral d′ was no longer significant (p = .55), indicating that anticipatory attentional orienting, rather than baseline memory accuracy, explained individual differences in fluid intelligence. For the retro-cue condition, neutral d′ also significantly predicted Raven’s scores, in Step 1, *β* = .82, p < .001, explaining 66.4% of the variance (*R*2 = .66). In Step 2, the addition of retro-cued d’ improved further the model, Δ*R*2 = .08, F(1, 38) = 12.53, p = .001, with retro-cued d’ emerging as a significant independent predictor, *β* = .78, p = .001. Retro neutral d′ was no longer significant (p = .70). Although multicollinearity between neutral and cued trials in the retro-cue condition was elevated (VIF = 7.38; Tolerance = .14), the cued predictor remained robust. These results suggest that attentional orienting during maintenance contributes uniquely to fluid intelligence, though less strongly than encoding-related orienting.

## Discussion

The current study investigated the interaction between attentional orienting and VSTM in individuals with ID, compared to chronologically matched neurotypical adults. We hypothesised that individuals with ID would derive smaller cueing benefits in support of VSTM, compared to TDA, reflecting a broader inefficiency in voluntary attentional deployment. We further predicted, for the ID group, that this reduction would be most pronounced for retro-cues. Finally, we expected that individual differences in attentional orienting efficiency would explain unique variance in fluid reasoning, over and above baseline VSTM performance, across individuals with varying levels of intellectual functioning. Using the well-established Attentional Orienting Task, assessing external and internal attention (Griffin & Nobre, 2003; Shimi, Nobre, et al., 2014; Shimi & Scerif, 2017), we examined the distinct effects of attention allocation before encoding and during maintenance of information on VSTM.

As expected, the TDA group outperformed individuals with ID across all trial types (cued, neutral) and cue conditions (pre-cue, retro-cue), reflecting poorer external and internal attentional orienting and VSTM performance in the ID group. This pattern underscores widespread attentional and memory-related impairments in ID. Importantly, while the TDA group exhibited significant pre-cue and retro-cue benefits, individuals with ID showed no cueing benefits in either pre-cue or retro-cue conditions, contrary to our predictions of preserved pre-cue benefits. At the same time, overall discrimination performance in the ID group was relatively low, which may limit the extent to which cueing manipulations can produce observable behavioural benefits in this task. Critically, however, despite poorer baseline VSTM performance, informative spatial cues did not improve memory recognition, suggesting difficulty in using voluntary attentional orienting to support VSTM. Accordingly, the absence of cueing benefits in this group should be interpreted as reflecting difficulty in effectively deploying voluntary attentional orienting to support VSTM, rather than as evidence that such mechanisms are entirely absent. Nevertheless, consistent with our final hypothesis, individual differences in both external (pre-cue) and internal (retro-cue) attentional orienting significantly predicted fluid reasoning.

Results from the TDA group align with a substantial body of work showing that neurotypical adults efficiently deploy attention to both perceptual inputs and internal representations to facilitate VSTM (Astle et al., 2011; Griffin & Nobre, 2003; Matsukura et al., 2014; Nobre et al., 2004; Schmidt et al., 2002; Souza & Oberauer, 2016). Similarly, the larger cueing benefits during VSTM encoding and maintenance processes observed in TDA, compared to ID, also mirror developmental findings that show increasing use of voluntary visuospatial orienting with age (Astle et al., 2012; Shimi, Nobre, et al., 2014; Shimi & Scerif, 2015, 2017). Nevertheless, in past developmental studies, even young children, aged 6, show clear pre-cue benefits, whereas retro-cue benefits emerge more gradually (Shimi, Nobre, et al., 2014; Shimi & Scerif, 2015, 2017). The absence of both pre-cue and retro-cue benefits in our ID group suggests that voluntary attentional allocation may not be effectively deployed to support VSTM under the present task conditions and may reflect atypical or developmentally immature control processes.

Current findings corroborate prior evidence of impairments in selective attention (Kim et al., 2021; Merrill & O’Dekirk, 1994) and VSTM in ID (Henry & MacLean, 2002; Lanfranchi et al., 2009; Vicari et al., 1995). Critically, we demonstrate for the first time that limitations in the controlled allocation of visuospatial attention may prevent individuals with ID from using informative cues to improve VSTM performance. Our use of a contemporary single-probe recognition task (rather than span tasks) provides new evidence of poor encoding and maintenance of multiple-item visual arrays. In everyday complex environments, we are typically faced with multiple information and selecting relevant over irrelevant stimuli is essential for adaptive behaviour. Individuals with ID who struggle with this process are likely to experience cascading deficits that hinder VSTM and real-world functioning, including reduced independence, a key impairment domain in ID (García-Pintor et al., 2024).

Previous research has attributed poor VSTM performance in ID to inefficient encoding strategies and increased vulnerability to interference (Henry & MacLean, 2002; Lanfranchi et al., 2009). Our findings extend these proposals by suggesting that inefficiencies in both encoding and maintenance strategies may contribute to poor VSTM in ID. Notably, past adult (Makovski et al., 2008) and developmental studies have shown that retro-cues protect internal representations from interference and the ability to do so predicts VSTM capacity (Shimi, Nobre, et al., 2014). Here, individuals with ID emerged unable to recruit attention and benefit from this protective mechanism, potentially leading to detrimental interference effects in their VSTM.

Moreover, difficulties in maintaining bound representations (i.e., item-location conjunction) have been reported in ID-associated syndromes (Jarrold & Brock, 2012). Visuospatial attention in service of perception, i.e., external attention (Treisman & Gelade, 1980; Treisman & Zhang, 2006; Wheeler & Treisman, 2002) as well as visuospatial attention in service of VSTM, i.e., internal attention (Shimi & Scerif, 2022) serve as the “glue” by which item features are bound to their locations, allowing individuals to generate robust representations in VSTM, improving later item recall. Current findings suggest that deficits in both attentional systems may lead to fragile or incomplete VSTM representations in ID, hindering later successful recognition.

These behavioural impairments could be underlined by altered brain functioning in ID. Developmental neurophysiological findings have shown that the maturation and efficiency of the frontoparietal control network relate to selective, flexible attentional deployment in support of VSTM (Shimi, Kuo, et al., 2014). This network undergoes atypical structural and functional developmental trajectories in ID (Ma et al., 2021), suggesting further that the difficulties in VSTM observed in ID may originate from impaired attentional control mechanisms.

To better understand the link between voluntary attentional orienting and intellectual ability, we conducted correlation and regression analyses between Raven’s 2 and AOT performance across all participants. Both pre-cue and retro-cue performance significantly predicted fluid intelligence, even after controlling for baseline performance and age. These findings indicate that attentional mechanisms during both VSTM encoding and maintenance contribute meaningfully to individual differences in non-verbal reasoning. Thus, these results highlight the importance of top-down attention to individual differences in fluid intelligence. Importantly, attentional orienting during encoding emerged as a stronger predictor than during maintenance, suggesting that the ability to efficiently direct attention toward perceptually relevant information during encoding plays a particularly critical role in supporting higher cognitive abilities. This may reflect differences in the cognitive operations required to select relevant perceptual input vs attentionally reactivate stored information. Notably, encoding operations may be more efficient and consistent in the general population, whereas maintenance strategies vary more widely across individuals and groups, potentially explaining the weaker predictive value of retro-cue performance. These results support theoretical models proposing that fluid intelligence relies not only on storage capacity but also on the efficiency of attentional control during the selection and retention of information (Kane & Engle, 2002; Rueda, 2018). Strategic prioritisation has been proposed as a key process by which individuals extract value from limited memory resources (Allen et al., 2025), and our findings suggest that this process may be compromised in individuals with ID due to impaired attentional control.

Overall, these findings align with previous research emphasising the close relationship between attentional selection and higher-order cognition (Engle, 2002; Unsworth et al., 2014). Neurocognitive models propose that anticipatory attention during encoding relies on top-down control from prefrontal and parietal regions to prioritise relevant sensory input, whereas retrospective attention during maintenance involves reactivating stored representations, possibly through parietal and medial temporal lobe interactions (Van Ede & Nobre, 2023). The present results suggest that attention during encoding may be more tightly coupled to the mechanisms that support fluid reasoning, perhaps because it shapes the initial quality and organisation of mental representations. In contrast, retrospective selection may depend more on internal cues and may vary in its effectiveness across individuals, especially in populations with cognitive impairments. Taken together, these findings contribute to a growing body of evidence that attentional control, especially at the point of entry into memory, is a key mechanism linking perception, memory, and intelligence (Burgoyne & Engle, 2020). Altogether, the current findings reinforce the importance of considering attention and VSTM as interdependent mechanisms that jointly constrain intellectual functioning.

This study is, to our knowledge, the first attempt to address the interaction between attentional orienting and VSTM in ID using a trial-type and cue-condition design matched with neurotypical controls. Nevertheless, we note some limitations. First, we focused exclusively on endogenous (voluntary) attention, and it remains possible that individuals with ID could benefit from exogenous (stimulus-driven) cues, particularly during encoding, to facilitate VSTM. Bottom-up attention develops earlier than top-down attention (Amso & Scerif, 2015) and is often spared in ID (Kim et al., 2021), suggesting that salient cues may still capture attention effectively. However, evidence from developmental research shows that attentional capture alone may not suffice, and cognitive control processes may be needed to translate salience into memory benefits. Pedale et al. (2022) found that salience enhanced VSTM and metamemory in adults, but not in children, reflecting the need for mature cognitive control to strategically encode and monitor salient information. Given that such executive functions are often impaired in ID (Nader-Grosbois, 2014; Spaniol & Danielsson, 2022), individuals may notice salient information but still fail to integrate it effectively into VSTM. Yet, other findings suggest that children can prioritise valuable information in VSTM when tasks are sufficiently motivating and structured (Atkinson et al., 2019). This raises the possibility that individuals with ID may benefit from similar scaffolding to help translate perceptual cues into strategic encoding, thereby improving both VSTM and functional independence. Future work should investigate whether the use of such cues can help overcome the deficits observed here, particularly at the stage of initial encoding.

Second, our ID sample was clinically heterogeneous, as it was based on the availability of participants in the Foundation and consent forms. While a more homogeneous sample may yield different cueing patterns, we believe that our mechanism-oriented approach, focusing on attentional control as a shared vulnerability across ID populations, offers insights that transcend diagnostic boundaries and inform dimensional models of cognitive function in neurodevelopment.

The findings of this study provide a foundation for future work exploring how attentional mechanisms might be leveraged in intervention, contributing to the creation of more interactive and personalised learning tools to the needs of individuals with ID within the school settings. For instance, Augmented Reality (AR) tools that train visual attention and VSTM could be adapted to support these cognitive abilities in individuals with ID. Recent findings show promise for supporting attention in individuals with ID through targeted interventions. For instance, music therapy and pictorial illustrations have been shown to increase attention span in children with mild ID (Jacob et al., 2021). Similarly, VR-based attention training using eye tracking has produced promising improvements in attentional focus (Patti et al., 2024). Mechanistically grounded interventions targeting cue use, distractor suppression, or internal selection could further bolster independence skills and adaptive learning.

### Conclusions

The present study represents a first step toward integrating two lines of research, visuospatial selective attention and VTSM processes, in individuals with ID. It highlighted that individuals with ID did not show behavioural benefits from selectively orienting attention to encode and maintain the goal-relevant information to boost their poor VSTM. In neurocognitive development, attentional mechanisms enhance VSTM by prioritising relevant input and suppressing distractors (Shimi et al., 2015; Shimi, Kuo, et al., 2014; Van Ede & Nobre, 2023). Attentional dysfunction in ID exacerbates VSTM impairments while contributing to overall cognitive and functional deficits. These findings emphasise the need to jointly evaluate attention and memory processes when examining cognitive deficits in ID, as both processes are fundamental for effective learning. Understanding further this interaction in ID offers the ground for designing cognitive supports that target the attentional constraints of VSTM in ID.

## Additional File

The additional file for this article can be found as follows:

10.5334/joc.498.s1 Supplementary Material. Table S1 Mean accuracy rates for each AOT condition and trial type, and mean Raven’s 2 raw score, by Group.

## Data Availability

The data cannot be made publicly available due to the sensitive nature of the dataset and the associated ethical and legal constraints. The Cyprus National Bioethics Committee approved the study and consent procedures on the condition that data would be accessible only to the research team. In line with this approval, all informed consent forms specified that the data would remain confidential and restricted to the research team. In addition, the foundation facilitating access to participants with intellectual disability required that data confidentiality be strictly maintained.
