# Supplementary material for: When Attention Fails Memory: Voluntary Orienting Deficits and Visual Short-Term Memory in Intellectual Disability
Source: J Cogn. 2026 Apr 20;9(1):26. doi: 10.5334/joc.498 (PMC13109914; doi:10.5334/joc.498)
Supplement: Supplementary Material. — Table S1 Mean accuracy rates for each AOT condition and trial type, and mean Raven’s 2 raw score, by Group. [file joc-9-1-498-s1.pdf]

Supplementary Material

Table S1

Mean accuracy rates for each AOT condition and trial type, and mean Raven's 2 raw score, by Group

Measure	ID (Mean ± SD)	TDA (Mean ± SD)
pre-cued present	0.62 (0.30)	0.96 (0.07)
pre-cued absent	0.30 (0.32)	0.94 (0.07)
pre-neutral present	0.62 (0.30)	0.75 (0.16)
pre-neutral absent	0.28 (0.31)	0.78 (0.16)
retro-cued present	0.60 (0.28)	0.86 (0.12)
retro-cued absent	0.32 (0.35)	0.92 (0.09)
retro-neutral present	0.63 (0.28)	0.74 (0.18)
retro-neutral absent	0.24 (0.30)	0.76 (0.21)
Raven's 2	11.90 (5.27)	104.68 (13.41)
